# Fully independent validation of eleven prognostic scores predicting progression to critically ill condition in hospitalized patients with COVID-19

**DOI:** 10.1016/j.bjid.2024.103721

**Published:** 2024-02-06

**Authors:** Vinicius Lins Costa Mello, Pedro Emmanuel Alvarenga Americano do Basil

**Affiliations:** Instituto Nacional de Infectologia Evandro Chagas – Fundação Oswaldo Cruz, Rio de Janeiro, RJ, Brazil

**Keywords:** COVID-19, Prognosis, Prediction models, Critical status, Mortality

## Abstract

**Introduction:**

COVID-19 remains an important threat to global health and maintains the challenge of COVID-19 hospital care. To assist decision making regarding COVID-19 hospital care many instruments to predict COVID-19 progression to critical condition were developed and validated.

**Objective:**

To validate eleven COVID-19 progression prediction scores for critically ill hospitalized patients in a Brazilian population.

**Methodology:**

Observational study with retrospective follow-up, including 301 adults confirmed for COVID-19 sequentially. Participants were admitted to non-critical units for treatment of the disease, between January and April 2021 and between September 2021 and February 2022. Eleven prognostic scores were applied using demographic, clinical, laboratory and imaging data collected in the first 48 of the hospital admission. The outcomes of greatest interest were as originally defined for each score. The analysis plan was to apply the instruments, estimate the outcome probability reproducing the original development/validation of each score, then to estimate performance measures (discrimination and calibration) and decision thresholds for risk classification.

**Results:**

The overall outcome prevalence was 41.8 % on 301 participants. There was a greater risk of the occurrence of the outcomes in older and male patients, and a linear trend with increasing comorbidities. Most of the patients studied were not immunized against COVID-19. Presence of concomitant bacterial infection and consolidation on imaging increased the risk of outcomes. College of London COVID-19 severity score and the 4C Mortality Score were the only with reasonable discrimination (ROC AUC 0.647 and 0.798 respectively) and calibration. The risk groups (low, intermediate and high) for 4C score were updated with the following thresholds: 0.239 and 0.318 (https://pedrobrasil.shinyapps.io/INDWELL/).

**Conclusion:**

The 4C score showed the best discrimination and calibration performance among the tested instruments. We suggest different limits for risk groups. 4C score use could improve decision making and early therapeutic management at hospital care.

## Introduction

Coronavirus Disease 2019 (COVID-19) is a respiratory infection that may be from asymptomatic, to severe cases and death.^1^ Since its emergence in December 2019 up to December 2022, COVID-19 has accounted for more than 601 million cases and 6.4 million deaths worldwide and overwhelmed health systems around the world.^2^^,^^3^ The incidence behavior in waves established substantial pressure on health services, especially when crowded hospitals have no room for additional critically ill cases.^4^ The COVID-19 progression rate to critically ill condition was estimated to be 22.9 %.^5^ Additionally, the estimated risk of Intensive Care Unit (ICU) admission, mechanical ventilation and overall mortality were 10.96 %, 7.1 % and 5.6 % respectively.^5^

After effective population vaccination, there was a decrease in the number of admissions in critical units and death.^6^ It is possible to see differences in time trends of hospitalizations, critical care admissions, and deaths from COVID-19 over the course of the pandemic. In adults over 50-years, there was a lower relative risk of Intensive Care Unit (ICU) admission of 23.3 % and 24.3 % when comparing the peaks of COVID-19 by Ômicron vs. Alpha and Ômicron vs Delta, respectively. When comparing the Ômicron to previous waves, deaths and ICU admissions were 4.5% vs. 21.3 % and 1% vs.4.3 % respectively,^7^ changing to a profile of high dissemination and a decreasing number of hospitalizations and deaths.^6^

Patients affected by moderate or extensive clinical presentation who seek hospital care make up the risk group for critical COVID-19. Early identification of patients at higher risk groups for disease progression at the emergency room or at hospital admission could aid decision making and improve individual and public health resources. One of the ways to help in this process would be through diagnostic and prognostic instruments at different levels of health care.^1^ Prognostic models, scoring systems and prediction tools were developed, validated and used in different health services around the world, for the early identification of potentially serious or critical patients.^1^^,^^4^^,^^8^, ^9^, ^10^, ^11^, ^12^, ^13^, ^14^, ^15^, ^16^

Studies have demonstrated promising applicability of some COVID-19 prognostic scores.^17^ A prognostic score for in-hospital death with Brazilian participants estimated 20.3 % mortality, later validated at Barcelona, Spain.^18^ Nevertheless, validation studies of prognostic scores with the Brazilian population are scarce. The aim of this study was to validate different prognostic instruments to predict COVID-19 progression to a severe condition in a fully independent sample of Brazilian patients.

## Methods

### Ethics

The study was developed in accordance with the Regulatory Guidelines and Standards for Research involving Human Beings (Resolution CNS/MS nº 466/2012). All participants signed a written consent. The Ethics Committee of the INI-Fiocruz registry and approval can be accessed at https://plataformabrasil.saude.gov.br/login.jsf with number CAAE 39,520,820.7.0000.5262.

### Source data and settings

This is a retrospective observational follow-up study carried out at Niterói/Rio de Janeiro, Brazil. All patients hospitalized at Hospital Santa Martha from January 1, 2021 to April 30, 2021 and all patients hospitalized at Hospital Niterói D'Or from September 1, 2021 to February 28, 2022 were sequentially included. In the 1st quarter of 2021, Alpha strain was the main circulating variant of the SARS-CoV-2 virus. There was little vaccine coverage at this time, high incidence rate of COVID-19 and high death rates from the disease.^6^ In July‒Oct/2021 a first wave of new cases caused by the Delta variant was observed. Later, a predominantly Ômicron variant wave was observed, regardless of vaccine increased coverage. Special groups were already receiving booster doses, with a gradual reduction in hospitalization rates in critical units and of mortality at this time.^6^^,^^7^^,^^19^

### Study participants

The inclusion criteria were: adult patients (18-years old or more); a positive RT-PCR result for COVID-19, obtained from respiratory swab or viable biological material representing active disease, collected between 3 and 10 days onset of symptoms, at any time during hospitalization; patients with a history of exposure, clinical findings or radiological image compatible with COVID-19 according to Ministry of Health criteria's at that time;^20^ patients with a completed hospitalization guide, allocated in non-critical sectors. The exclusion criteria were absence of clinical evaluation in the first 48 h; discharge or death before completing 24 h of hospitalization; critical conditions at admission or directly admitted to intensive support units. Critical conditions were considered as: 1-Glasgow coma scale <8; 2-Need to use vasoactive amines; 3-Need intubation and mechanical ventilation support; 4-Need for acute dialysis therapy.

### Criteria and measurement data

The predictors' assessments were performed at hospital admission, eventually considered up to 48 h after admission. Patients were submitted to a protocol where: 1-Clinical evaluation, seeking to identify relevant clinical elements (e.g., fever, headache, coryza, sore throat, myalgia, dry cough, risk of exposure); 2-Laboratory tests; and 3-Chest image by computerized tomography.

The criteria used for hospital admission sectors at the time were in accordance with the parameters defined by Brazilian Ministry of Health, which were: 1-Moderate cases: patient with clinical or radiological evidence of respiratory disease and SatO2 ≥ 94 % in room air; 2-Severe cases: patient with respiratory rate > 30 ibm, SatO2 < 94 % on room air (or, in patients with chronic hypoxia, *a* > 3 % reduction from baseline), PaO2/FiO2 ratio 〈 300 mmHg, or opacities in 〉 50 % of the lung.^20^ Standard treatment was offered according to each hospital's protocol based on guidelines at the time.

### Prediction models

The following prediction instruments were applied: 1-COVID-GRAM Critical Illness Risk Score;^14^ 2-Multilobular infiltration, hypo-Lymphocytosis, Bacterial coinfection, Smoking history, hyper-Tension and Age (MuLBSTA) Score,^10^ 3-Comorbidity, Age, Lymphocyte and LDH (CALL) score;^12^ 4-ABC-GOALS score;^15^ 5-Quick COVID-19 severity index (qCSI);^11^ 6-Kuwait Progression Indicator (KPI) score;^8^ 7-College of London COVID-19 severity score (Cols)^4^; 8-Prognostic Index for Clinical Deterioration;^9^ 9-Severity COVID-19 Risk Prediction Model;^1^ 10-ANDC score;^16^ 11–4C Mortality score.^13^

### Outcomes

In this study, the composite outcome of greatest interest was admission to the intensive care unit or death, the same used in most prediction models studied, such as the COVID-GRAM Critical Illness Risk Score,^14^, MuLBSTA Score^10^, CALL score,^12^ ABC-GOALS score,^15^ KPI score,^8^ Cols,^4^ Prognostic Index for Clinical Deterioration,^9^ Severity COVID-19 Risk Prediction Model,^1^ ANDC score.^16^ Outcomes for each score were considered as defined in the respective original report, such as the need for invasive ventilation,^1^^,^^14^ clinical respiratory deterioration,^11^^,^^12^ length of stay,^12^ and prospective analysis of outcomes^1^^,^^4^^,^^10^ and death up to 28-days after admission.^13^

### Potential predictors for the outcomes

Potential risk predictors for COVID-19 progression among the models studied was quite heterogeneous, ranging from 4 to 17, and containing more or less frequent demographic, clinical, laboratory and imaging elements. The common predictors present in 4 or more of the scores studied were: (a) Demographics (age,^1^^,^^4^^,^^8^, ^9^, ^10^^,^^12^, ^13^, ^14^^,^^16^ sex,^1^^,^^4^^,^^13^^,^^15^ presence and/or number of comorbidities^1^^,^^4^^,^^9^^,^^10^^,^^12^, ^13^, ^14^, ^15^); (b) Clinical (dyspnea or respiratory rate,^1^^,^^4^^,^^9^^,^^11^^,^^13^, ^14^, ^15^ peripheral oxygen saturation^4^^,^^9^^,^^11^^,^^13^); (c) Laboratory (CRP,^1^^,^^4^^,^^8^^,^^13^^,^^16^, LDH,^9^^,^^12^^,^^14^^,^^15^, number of lymphocytes^8^, ^9^, ^10^^,^^12^^,^^14^^,^^16^); and (d) Radiological changes,^4^^,^^9^^,^^10^^,^^14^^,^^15^ which allowed its collection and better application.

On the other hand, there were less frequent and less available predictors in the context of hospital admission and, therefore, more difficult to apply in our analysis, such as hemoptise,^14^ smoking and bacterial co-infection,^10^ ethnicity,^4^ interleukin-6, ferritin and fibrinogen.^9^ There were also instruments that used other existing scores for their application, such as the Charlson score^15^ and the RALE score^4^ adapted to COVID-19. Data were collected from medical records and consulting assistant health professionals.

Data was extracted from medical records to an electronic standard data collection instrument by one of the authors (VLCM) and an undergrad trainee supervised by the second author (PEAAB). There was some training in data extraction and research forms improvement at the beginning. The extractors were not blinded to the research hypothesis, and no extractors interrater agreement was estimated.

### Data analysis

The outcome prevalence estimated from administrative data before the study ranged from 50 % to 72 % depending on period and health unit. Therefore, we assumed that 300 subjects would be enough to reach the 100 subjects with events and the 100 subjects without the events.^21^ For prediction purposes, missing data was imputed with multiple imputation procedures using the CART models with “mice” R package.

Data analysis was conducted in R software following the steps: description of possible predictors to be explored; exploration of missing data patterns and the need for data imputation; verification of the need for recoding of the predictors; and validation (discrimination and calibration) of the different scores. The different prediction instruments were tested with the same data, reproducing the original model or using the original recommended scores in the population of interest. The validity measures used to measure discrimination were area under the ROC curve and R squared. Additionally, for the calibration measures the calibration belt, model's intercept and slope and predictions errors were used (average, maximum and percentile 90).^22^ Additionally, decision limits were estimated with the “uncertain interval” method^23^ in order to allow different courses of action, for example, (a) Low risk recommending discharge, (b) Moderate risk recommending monitoring, (c) High risk recommending early transfer to critical care.

## Results

About half of 301 participants included and analyzed came from each health unit, Hospital Santa Martha and Hospital Niterói D'Or. The composite outcome overall prevalence was 41.86 %, 56.96 % at Niteroi D'Or, 26.67 % at Santa Martha hospital (Table 1). The overall mortality was 16.61 %, 15.23 % at Niteroi D'Or and 18.00 % at Santa Martha hospital. Median age is higher, and males are more frequent in the outcome group. Most participants were not immunized or data regarding immunization was not available. Among those vaccinated, most hospitalized patients had been immunized with CoronaVac. Most participants who had worse respiratory parameters profile progressed to the outcome more frequently, most evidently those with worse saturation (Table 1).

**Table 1:** Clinical, demographic characteristics, signs and symptoms by composite outcome.

	**No**	**Yes**	**Total**
**Total**	175	126	301
**Inpatient unit**			
Niteroi D'Or	65 (37.14)	86 (68.25)	151 (50.17)
Santa Martha	110 (62.86)	40 (31.75)	150 (49.83)
**Age at admission**			
Median (IQR)	63.00 (47.50‒72.00)	70.50 (62.00‒82.75)	66.00 (53.00‒76.00)
**Sex at birth**			
Male	82 (46.86)	71 (56.35)	153 (50.83)
Female	93 (53.14)	55 (43.65)	148 (49.17)
**Smoking**			
No	91 (91.92)	38 (65.52)	129 (82.17)
Past	5 (5.05)	18 (31.03)	23 (14.65)
Current	3 (3.03)	2 (3.45)	5 (3.18)
**Ethnicity**			
White	19 (86.36)	21 (87.50)	40 (86.96)
Not white	3 (13.64)	3 (12.50)	6 (13.04)
**Immunization**			
Not immunized/Not informed	84 (57.14)	28 (25.23)	112 (43.41)
Coronavac	25 (17.01)	44 (39.64)	69 (26.74)
Astrazeneca	27 (18.37)	32 (28.83)	59 (22.87)
Pfizer	9 (6.12)	4 (3.60)	13 (5.04)
Janssen	2 (1.36)	3 (2.70)	5 (1.94)
**Immunization scheme**			
Partial	13 (20.63)	15 (18.07)	28 (19.18)
Complete	50 (79.37)	68 (81.93)	118 (80.82)
**Weight (Kg)**			
Median (IQR)	75.00 (66.00‒88.00)	79.00 (68.00‒87.50)	76.00 (67.00‒88.00)
**Height (m)**			
Median (IQR)	1.68 (1.61‒1.75)	1.68 (1.60‒1.73)	1.68 (1.60‒1.74)
**Body mass index (Kg/m^2^)**			
Median (IQR)	27.06 (23.62‒30.49)	27.04 (23.98‒31.08)	27.06 (23.89‒30.86)
**Symptoms days on admission**			
Median (IQR)	8.47 (6.03‒10.59)	7.47 (3.83‒9.93)	7.78 (5.46‒10.48)
**Hemoptysis**			
No	174 (99.43)	122 (99.19)	296 (99.33)
Yes	1 (0.57)	1 (0.81)	2 (0.67)
**Dyspnea**			
No	76 (43.68)	44 (35.20)	120 (40.13)
Yes	98 (56.32)	81 (64.80)	179 (59.87)
**Respiratory frequency**			
[12, 20]	155 (94.51)	94 (78.33)	249 (87.68)
[20, 30]	9 (5.49)	26 (21.67)	35 (12.32)
**Pulse oximetry (0‒100 %)**			
Median (IQR)	95.00 (93.00‒97.00)	93.00 (89.00‒96.00)	95.00 (92.00‒97.00)
**Pulse oximetry (0‒100 %)**			
[55, 92]	29 (17.68)	57 (45.97)	86 (29.86)
[92, 100]	135 (82.32)	67 (54.03)	202 (70.14)
**Oxygen flow (L/min)**			
[0, 4]	90 (81.08)	82 (86.32)	172 (83.50)
[4, 9]	19 (17.12)	12 (12.63)	31 (15.05)
(9, 15]	2 (1.80)	1 (1.05)	3 (1.46)
**Systolic blood pressure (mmHg)**			
Median (IQR)	120.00 (120.00‒132.00)	123.00 (110.00‒140.00)	121.50 (116.00‒137.00)
**Systolic blood pressure (mmHg)**			
[68, 100]	11 (6.43)	18 (14.40)	29 (9.80)
[100, 140]	139 (81.29)	79 (63.20)	218 (73.65)
[140, 216]	21 (12.28)	28 (22.40)	49 (16.55)

IQR, Interquartile Range.

The most prevalent comorbidities were systemic arterial hypertension and diabetes mellitus. There is an apparent positive linear effect between the number of comorbidities and a higher risk of outcome, reaching 67 % in patients with 3 or more comorbidities. Likewise, the higher the Charlson score, the greater the risk of outcome (Table 2).

**Table 2:** Comorbidities by composite outcome.

	**No**	**Yes**	**Total**
**Total**	175	126	301
**Systemic arterial hypertension**			
No	84 (48.00)	44 (34.92)	128 (42.52)
Yes	91 (52.00)	82 (65.08)	173 (57.48)
**Diabetes**			
No	129 (73.71)	71 (56.35)	200 (66.45)
Yes	46 (26.29)	55 (43.65)	101 (33.55)
**Obesity**			
BMI <30	140 (80.00)	93 (73.81)	233 (77.41)
BMI ≥30	35 (20.00)	33 (26.19)	68 (22.59)
**COPD**			
No	167 (95.43)	113 (89.68)	280 (93.02)
Yes	8 (4.57)	13 (10.32)	21 (6.98)
**Coexistence of coronary heart disease**			
No	160 (91.43)	103 (83.06)	263 (87.96)
Yes	15 (8.57)	21 (16.94)	36 (12.04)
**History of cancer**			
No	168 (96.00)	101 (80.80)	269 (89.67)
Yes	7 (4.00)	24 (19.20)	31 (10.33)
**Chronic cardiopathy**			
No	166 (94.86)	101 (80.16)	267 (88.70)
Yes	9 (5.14)	25 (19.84)	34 (11.30)
**Chronic kidney disease**			
No	170 (97.70)	114 (90.48)	284 (94.67)
Yes	4 (2.30)	12 (9.52)	16 (5.33)
**Cerebrovascular disease**			
No	171 (97.71)	109 (86.51)	280 (93.02)
Yes	4 (2.29)	17 (13.49)	21 (6.98)
**Hepatitis B**			
No	175 (100.00)	126 (100.00)	301 (100.00)
**Immunodeficiency**			
No	166 (94.86)	109 (86.51)	275 (91.36)
Yes	9 (5.14)	17 (13.49)	26 (8.64)
**Liver disease**			
No	171 (98.84)	123 (97.62)	294 (98.33)
Yes	2 (1.16)	3 (2.38)	5 (1.67)
**Asthma**			
No	166 (95.40)	121 (96.03)	287 (95.67)
Yes	8 (4.60)	5 (3.97)	13 (4.33)
**Chronic lung disease**			
No	168 (96.00)	109 (86.51)	277 (92.03)
Yes	7 (4.00)	17 (13.49)	24 (7.97)
**HIV infection**			
No	174 (99.43)	126 (100.00)	300 (99.67)
Yes	1 (0.57)	0 (0.00)	1 (0.33)
**Malignancy for at least 6-months**			
No	172 (98.29)	106 (84.13)	278 (92.36)
Yes	3 (1.71)	20 (15.87)	23 (7.64)
**Number of comorbidities**			
0	56 (32.18)	15 (12.20)	71 (23.91)
1	53 (30.46)	22 (17.89)	75 (25.25)
2	39 (22.41)	33 (26.83)	72 (24.24)
≥3	26 (14.94)	53 (43.09)	79 (26.60)
**Charlson Comorbidity Index**			
Median (IQR)	2.00 (1.00‒4.00)	5.00 (3.00‒8.00)	3.00 (1.00‒6.00)
**Charlson Comorbidity Index**			
0	35 (20.11)	8 (6.61)	43 (14.58)
1	28 (16.09)	8 (6.61)	36 (12.20)
2	34 (19.54)	15 (12.40)	49 (16.61)
3	28 (16.09)	14 (11.57)	42 (14.24)
4	16 (9.20)	13 (10.74)	29 (9.83)
≥5	33 (18.97)	63 (52.07)	96 (32.54)

BMI, Body Mass Index; COPD, Chronic Obstructive Pulmonary Disease; IQR, Interquartile Range.

There was no relevant C-Reactive Protein (CRP) nor Urea relationship with the outcome. On the other hand, patients with d-dimer elevation had a slightly higher risk of a composite outcome than those without d-dimer elevation (Table 3). Likewise, relevant differences in the risk of occurrence of the outcome was observed among participants who had elevated procalcitonin or concomitant bacterial infection with COVID-19 (Table 3).

**Table 3:** Laboratory results by composite outcome.

	**No**	**Yes**	**Total**
**Total**	175	126	301
**RDW (%)**			
Median (IQR)	13.00 (12.50‒13.80)	13.60 (12.70‒14.70)	13.20 (12.60‒14.10)
**Leukocyte count (×10^9/L)**			
Median (IQR)	7160.00 (5180.00‒9030.00)	7370.00 (5512.50‒9975.00)	7290.00 (5360.00‒9250.00)
**Leukocytosis**			
No	147 (84.48)	95 (75.40)	242 (80.67)
Yes	27 (15.52)	31 (24.60)	58 (19.33)
**Neutrophil count (×10^9/L)**			
Median (IQR)	5168.00 (3685.00‒7068.00)	5674.00 (3754.25‒7681.00)	5340.00 (3694.00‒7428.50)
**Lymphocyte count (×10.9/L)**			
Median (IQR)	1247.00 (862.00‒1672.00)	1033.00 (787.50‒1585.75)	1165.00 (830.00‒1642.00)
**Lymphocyte percentage (0‒100 %)**			
Median (IQR)	19.09 (11.01‒25.01)	15.01 (10.25‒20.90)	16.01 (11.00‒24.00)
**Lymphopenia (×10^9/L)**			
No	118 (67.82)	68 (53.97)	186 (62.00)
Yes	56 (32.18)	58 (46.03)	114 (38.00)
**Neutrophils/lymphocytes ratio**			
Median (IQR)	3.70 (2.67‒6.99)	5.06 (3.32‒8.04)	4.66 (2.87‒7.27)
**Monocyte count (×10^9/L)**			
Median (IQR)	348.00 (270.00‒496.00)	394.50 (245.50‒574.75)	376.00 (253.50‒528.50)
**Monocyte percentage (0‒100 %)**			
Median (IQR)	5.01 (4.00‒7.00)	5.15 (4.00‒7.57)	5.01 (4.00‒7.00)
**Glucose (mg/dL)**			
Median (IQR)	118.00 (103.00‒146.00)	130.50 (110.25‒174.50)	123.00 (105.50‒158.00)
**C-reactive protein ‒ CRP (mg/L)**			
Median (IQR)	39.00 (5.05‒97.00)	8.85 (4.30‒50.25)	21.40 (4.30‒82.00)
**CRP elevation**			
No	44 (25.58)	39 (30.95)	83 (27.85)
Yes	128 (74.42)	87 (69.05)	215 (72.15)
**D-dimer elevation**			
No	35 (20.47)	18 (14.40)	53 (17.91)
Yes	136 (79.53)	107 (85.60)	243 (82.09)
**D-dimer (mg/mL)**			
Median (IQR)	1.20 (0.60‒480.00)	562.00 (2.25‒1205.90)	2.85 (0.78‒735.20)
**Lactic dehydrogenase ‒ LDH (U/L)**			
Median (IQR)	413.00 (235.00‒626.00)	334.50 (225.50‒522.00)	375.00 (230.00‒555.50)
**LDH elevation**			
No	63 (50.00)	28 (32.94)	91 (43.13)
Yes	63 (50.00)	57 (67.06)	120 (56.87)
**Urea (mg/dL)**			
Median (IQR)	34.00 (25.50‒43.00)	44.50 (30.00‒74.75)	36.00 (27.00‒52.00)
**Creatinine (mg/dL)**			
Median (IQR)	1.05 (0.80‒1.27)	1.10 (0.78‒1.42)	1.09 (0.80‒1.35)
**Creatinine elevation**			
No	94 (54.02)	58 (46.40)	152 (50.84)
Yes	80 (45.98)	67 (53.60)	147 (49.16)
**AST (U/L)**			
Median (IQR)	36.00 (30.00‒55.00)	41.00 (27.25‒61.00)	37.50 (29.00‒56.25)
**AST elevation**			
No	107 (76.43)	64 (67.37)	171 (72.77)
Yes	33 (23.57)	31 (32.63)	64 (27.23)
**Direct bilirubin (mg/dL)**			
Median (IQR)	0.30 (0.20‒0.50)	0.40 (0.20‒0.80)	0.40 (0.20‒0.60)
**Direct bilirubin (mmoL/L)**			
Median (IQR)	24.00 (4.14‒24.00)	5.52 (3.45‒9.66)	5.86 (3.84‒24.00)
**Procalcitonin (ng/mL)**			
Median (IQR)	0.10 (0.06‒0.11)	0.12 (0.08‒0.28)	0.10 (0.07‒0.18)
**Procalcitonin elevation**			
No	111 (99.11)	65 (87.84)	176 (94.62)
Yes	1 (0.89)	9 (12.16)	10 (5.38)
**Troponin type**			
Conventional	2 (4.55)	0 (0.00)	2 (1.89)
Ultra-sensitive	42 (95.45)	62 (100.00)	104 (98.11)
**High sensitivity troponin (ng/L)**			
Median (IQR)	3.22 (1.50‒9.40)	9.48 (2.18‒29.43)	5.73 (1.50‒17.87)
**Serum ferritin (ng/mL)**			
Median (IQR)	729.50 (309.00‒1057.20)	498.00 (300.00‒1154.00)	562.00 (304.00‒1085.60)
**Serum ferritin elevation**			
No	58 (56.31)	17 (43.59)	75 (52.82)
Yes	45 (43.69)	22 (56.41)	67 (47.18)
**PO2 (S) (mmHg)**			
Median (IQR)	75.00 (59.50‒99.00)	90.50 (69.25‒144.75)	84.00 (65.00‒125.00)
**FiO2 (F) (%)**			
Median (IQR)	94.00 (48.00‒97.00)	43.50 (40.00‒97.00)	70.00 (40.00‒97.00)
**S/F**			
Median (IQR)	84.38 (67.55‒135.10)	179.75 (120.20‒321.88)	134.85 (83.46‒263.54)
**Concomitant bacterial infection**			
No	152 (92.12)	99 (82.50)	251 (88.07)
Yes	13 (7.88)	21 (17.50)	34 (11.93)

RDW, Red Cell Distribution Width; CRP, C-Reactive Protein; LDH, Lactic Dehydrogenase; AST, Aspartate Aminotransferase; PO2, Partial Pressure of Oxygen; FiO2, Inspired Fraction of Oxygen; IQR, Interquartile Range.

There is an apparent positive linear relationship of the composite outcome with the Radiological RALE Score index. There is also a higher risk of occurrence of the outcome in 65 % of the participants with pulmonary consolidation. The tomographic analysis of the pulmonary involvement less versus equal or greater than 50 % showed a difference in the risk of a composite outcome (Table 4).

**Table 4:** Image findings by composite outcome.

	**No**	**Yes**	**Total**
**Total**	175	126	301
**Radiological RALE Score (0‒8)**			
0	18 (10.59)	17 (13.71)	35 (11.90)
1	29 (17.06)	9 (7.26)	38 (12.93)
2	87 (51.18)	45 (36.29)	132 (44.90)
3	26 (15.29)	25 (20.16)	51 (17.35)
4	9 (5.29)	12 (9.68)	21 (7.14)
5	0 (0.00)	1 (0.81)	1 (0.34)
6	1 (0.59)	13 (10.48)	14 (4.76)
8	0 (0.00)	2 (1.61)	2 (0.68)
**Image findings**			
Normal	6 (3.51)	2 (1.60)	8 (2.70)
No specific signs	22 (12.87)	23 (18.40)	45 (15.20)
Frosted glass opacity	133 (77.78)	81 (64.80)	214 (72.30)
Consolidation	10 (5.85)	19 (15.20)	29 (9.80)
**Multilobar infiltrates**			
No	18 (10.59)	17 (13.71)	35 (11.90)
Yes	152 (89.41)	107 (86.29)	259 (88.10)
**Chest CT with 50 % or more involvement**			
No	160 (94.12)	96 (77.42)	256 (87.07)
Yes	10 (5.88)	28 (22.58)	38 (12.93)

CT, Computed Tomography.

**Fig. 1:**
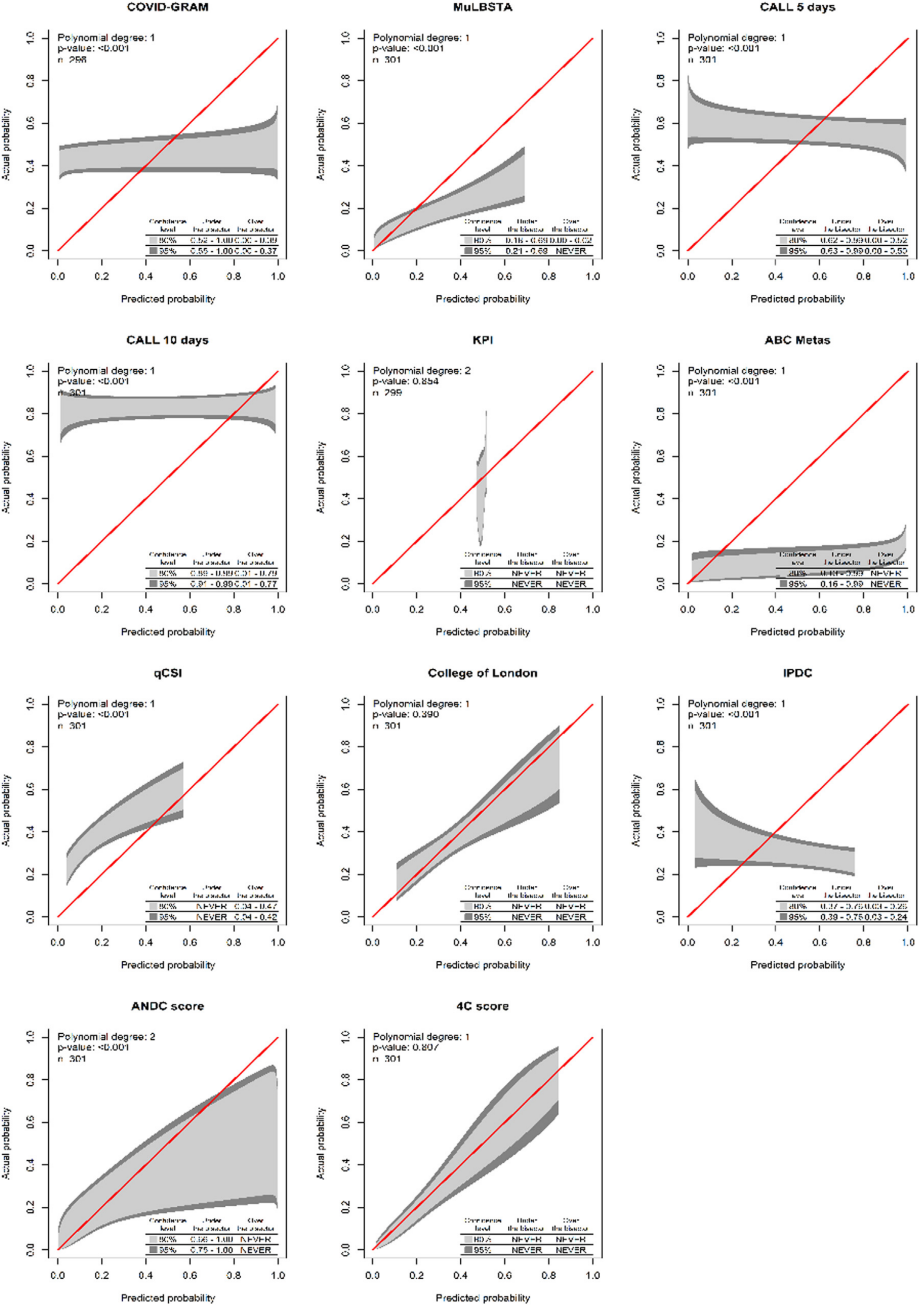
All Scores calibration belt.

Only two instruments showed acceptable calibration (Fig. 1) and discrimination: the College of London Score (Cols) and the 4C score. The latter is the one with the best discrimination and calibration performance (Table 5). Additionally, for the 4C score only, the upper and lower decision threshold of the uncertain interval was estimated as 0.239 and 0.318 respectively. Therefore, predictions below 0.230 should be considered as at lower risk of progression to critically ill condition, predictions between 0.318 are not able to discriminate between those at high and low risk of progression, and predictions above 0.318 should be considered as at higher risk of disease progression. As our latest result, we provide a web tool of the 4C Mortality score with the updated limits and risk classification for the Brazilian population (https://pedrobrasil.shinyapps.io/INDWELL/).

**Table 5:** Comparative performance of prediction scores for COVID-19 severity.

**Score**	**ROC AUC**	**R2**	**Intercept**	**Slope**	**Emax**	**E90**	**Eavg**
COVID-GRAM	0.535	−11.732	−0.174	0.038	0.543	0.501	0.357
MuLBSTA	0.712	−0.073	−1.031	0.533	0.306	0.306	0.132
CALL 5 days	0.449	−1.257	0.31	−0.078	0.678	0.428	0.315
CALL 10 days	0.502	−4.15	1.616	0.032	0.838	0.722	0.345
KPI	0.561	0.007	0.092	2.356	0.211	0.125	0.044
ABC goals	0.593	−196.443	−2.3	0.217	0.97	0.778	0.625
qCSI	0.669	−0.239	0.287	0.499	0.182	0.182	0.159
Cols	0.647	0.085	−0.163	0.761	0.163	0.076	0.05
IPDC	0.453	−2.347	−0.874	−0.177	0.503	0.503	0.414
MPR composite outcome	0.573	−0.308	−0.785	0.177	0.536	0.277	0.137
MPR composite death	0.631	−0.251	−1.609	0.265	0.545	0.195	0.084
4C score	0.798	0.277	−0.014	1.066	0.273	0.057	0.026
ANDC score	0.543	−897.989	−1.61	−0.001	0.838	0.838	0.405

COVID-GRAM Critical Illness Risk Score; MuLBSTA score, Multilobular infiltration, hypo-Lymphocytosis, Bacterial coinfection, Smoking history, hyper-Tension and Age; CALL score, Comorbidity, Age, Lymphocyte and LDH; ABC-GOALS score; Qcsi, Quick COVID-19 severity index; KPI score, Kwait Progression Indicator; COLS, College of London COVID-19 severity score; IPDC, Prognostic Index for Clinical Deterioration; MPR, Severity COVID-19 Risk Prediction Model; ANDC score; 4C Mortality score.

## Discussion

The main results to be discussed are: (a) Some biomarkers that usually are considered clinically relevant were tested as predictors in very few instruments (e.g., concomitant bacterial infection, d-dimer and procalcitonin); (b) There is a positive relationship with comorbidities or the number of comorbidities and the risk to progress to critical condition; (c) There is a positive relationship between the degree of pulmonary radiological involvement and the higher risk of occurrence of the outcome studied; (d) The 4C prognostic score was the one with the highest performance with reasonable applicability, good enough to be recommended for this population; (e) It was possible to estimate decision thresholds to recommend different courses of action for this population using the 4C score.

From the beginning of the pandemic, aspects regarding dissemination, lethality, mass prevention measures, among others, varied significantly. The advent of vaccines and the occurrence of viral variants are examples of these changes. Throughout the pandemic, a worldwide effort was made to develop vaccines against COVID-19.^6^^,^^19^ After vaccines became available, there was a reduction in the frequency of severe disease, ICU admission and mortality,^24^ changing the course of the pandemic.

In our study, participants who received the Chinese CoronaVac vaccine were the most frequent. Additionally, these participants were also the elders and the ones with the outcome more frequently observed. In Brazil, this was the first vaccine available for the public.^25^ Protection against severe disease presentation due to viral variants remained substantial, although age and multiple comorbidities contribute to worse outcomes.^26^ The considerable large time periods in the inclusion of participants probably allowed the inclusion of participants with different SARS-CoV-2 variants and participants from a population with different degrees of vaccination. One must consider that the vaccination and different strains may change the applicability of the prediction instruments only if the mortality and the critical illness incidence relationship with the presence or absence of the predictors also change.^6^^,^^7^^,^^24^ That is, once the patient is admitted to the emergency room and a prediction instrument would be applied, either the choice of the instrument is influenced by these information, or the instrument should adjust the prediction given these information. However, none of the instruments considered any of these limitations. Additionally, mortality was essentially the same in both vaccinated and non-vaccinated groups, and composite outcome was twice as frequent in the vaccinated group. When we estimate the 4C prediction instrument performance stratified by vaccination status, the discrimination performance is slightly better amongst the vaccinated, although slightly less calibrated (data not shown). Unfortunately, there is no data regarding the variants to extend such discussion. Therefore, the evidence suits reasonably for a population of hospitalized COVID-19 patients regardless of vaccination status.

We observed a substantial difference in the outcome prevalence among patients who had a concomitant bacterial infection with COVID-19. This infectious association would likely increase clinical compromise (systemic and cardiorespiratory) as well create doubts regarding correct antibiotics use. Only one score has included this variable to predict COVID-19 severity progression.^10^

D-dimer and other hematological changes are important features and significantly increase with disease severity.^27^ In multivariable analyses, death was associated with increased d-dimer.^27^ Elevated d-dimer over time was observed in nonsurvivors compared with more stable levels in survivors.^28^ In our study, patients with d-dimer elevation also had a slightly higher risk of a composite outcome than those without d-dimer elevation.

Multiple comorbidities and underlying conditions have been associated with COVID-19 to severe illness, with a higher prevalence of hospitalization, ICU admission and mechanical ventilation, or death.^29^ We also observed a positive linear relationship between the number of comorbidities and the risk of the outcomes, either through the number of comorbidities or the Charlson Score.

There are several imaging patterns of pulmonary involvement.^30^ In this study, we observed that radiographic involvement in patients with COVID-19 is a fundamental element in predicting disease severity. However, there is a 48 % prevalence of outcome in the “0” RALE index group. This may be associated with the presence of other predictors that may not have respiratory findings that add risk to the individual. We also observed that the presence of pulmonary consolidation alone would already indicate a risk of outcome of 65 % and a pulmonary involvement of 50 % or more has twice the risk of outcome. ARDS was estimated to occur in 20 % of the COVID-19 patients, and mechanical ventilation was implemented in 12.3 % of them.^28^ In the United States, 12 % to 24 % of hospitalized patients with altered respiratory symptoms progressed to mechanical ventilation.^29^

In the 4C Mortality Score^13^ four risk groups were defined with corresponding mortality rates determined: low risk (0‒3 score, mortality rate 1.2 %), intermediate risk (4‒8 score, mortality rate 9.9 %), high risk (9‒14 score, mortality rate 31.4 %), and very high risk (≥ 15 score, mortality rate 61.5 %). However, these groups seemed to be arbitrarily defined. The original recommended course of action for each risk group are: patients within the low risk groups could be suitable for management in the community; the intermediate risk group could be suitable for ward level monitoring; patients within high or very high could start promptly treatment and early escalation to critical care, if appropriate.^13^ However, in our population the risk distribution seems to be different from the 4C original population. Therefore, it is reasonable to adapt the risk classification to this population. As only three courses of action are recommended, we divided the estimated risk of outcome (not the intermediate score) into three categories (low risk, intermediate risk and high risk) allowing similar interpretation.

In this study, we had some limitations, such as the large number of missing data for some predictors, mainly those from the laboratory. Although the performance of the instruments can be estimated with imputed data, the lack of availability of certain predictors also opens an applicability and inference discussion. The different protocols for the management of COVID-19 cases in the two target hospitals of this study may influence the outcome incidence in different directions, as they had different clinical, structural or administrative criteria for directing patients to critical sectors. This could be in the way of patients with indication for admission to the critical unit, who remained in non-critical beds due to the smallest number of ICU beds. Finally, rapid changes in the epidemic with the emergence of new strains, the advent of vaccines and other preventive and treatment measures may have changed the populations characteristics in a way, that is the relationship of the clinical findings with the critical illness incidence, that score performance could require updating often. Even if the performance verified here is an “average” of the observed scenarios, attention must be paid to whether the future scenarios will continue to change, in such a way as to raise questions about the model's performance in the future.

## Conclusions

The validation of the prognostic models included here had very heterogeneous performance to predict critical illness and death, in patients already admitted to the emergency room or to non-critical units. The College of London COVID-19 severity score and the 4C Mortality Score showed the best discrimination and calibration performance. These findings are in accordance with validation in other populations, and we suggest different limits for risk groups.

## Conflicts of interest

The authors declare that they have no known competing financial interests or personal relationships that could have appeared to influence the work reported in this paper.

## References

[1] Woo S.H., Rios-Diaz A.J., Kubey A.A., Cheney-Peters D.R., Ackermann L.L., Chalikonda D.M. (2021). Development and validation of a web-based severe COVID-19 risk prediction model. Am J Med Sci.

[2] Gallo Marin B., Aghagoli G., Lavine K., Yang L., Siff E.J., Chiang S.S. (2021). Predictors of COVID-19 severity: a literature review. Rev Med Virol.

[3] Woolf S.H., Chapman D.A., Sabo R.T., Zimmerman E.B. (2021). Excess deaths from COVID-19 and other causes in the US, March 1, 2020, to January 2, 2021. JAMA.

[4] Galloway J.B., Norton S., Barker R.D., Brookes A., Carey I., Clarke B.D. (2020). A clinical risk score to identify patients with COVID-19 at high risk of critical care admission or death: an observational cohort study. J Infect.

[5] Li J., Huang D.Q., Zou B., Yang H., Hui W.Z., Rui F. (2021). Epidemiology of COVID-19: a systematic review and meta-analysis of clinical characteristics, risk factors, and outcomes. J Med Virol.

[6] Iuliano A.D., Brunkard J.M., Boehmer T.K., Peterson E., Adjei S., Binder A.M. (2022). Trends in disease severity and health care utilization during the early omicron variant period compared with previous SARS-CoV-2 high transmission periods ‒ United States, December 2020 – January 2022. MMWR Morb Mortal Wkly Rep.

[7] Abdullah F., Myers J., Basu D., Tintinger G., Ueckermann V., Mathebula M. (2022). Decreased severity of disease during the first global omicron variant covid-19 outbreak in a large hospital in tshwane, south africa. Int J Infect Dis.

[8] Al Youha S, Doi SA, Jamal MH, Almazeedi S, Al Haddad M, AlSeaidan M, et al. Validation of the Kuwait progression indicator score for predicting progression of severity in COVID19. medRxiv. 2020. 10.1101/2020.05.21.20108639

[9] Cecconi M., Piovani D., Brunetta E., Aghemo A., Greco M., Ciccarelli M. (2020). Early predictors of clinical deterioration in a cohort of 239 patients hospitalized for COVID-19 infection in Lombardy, Italy. J Clin Med.

[10] Guo L., Wei D., Zhang X., Wu Y., Li Q., Zhou M. (2019). Clinical features predicting mortality risk in patients with viral pneumonia: the MuLBSTA score. Front Microbiol.

[11] Haimovich A., Ravindra N.G., Stoytchev S., Young H.P., Wilson F.P., van Dijk D. (2020). Development and validation of the quick COVID-19 severity index (qCSI): a prognostic tool for early clinical decompensation. Ann Emerg Med.

[12] Ji D., Zhang D., Xu J., Chen Z., Yang T., Zhao P. (2020). Prediction for progression risk in patients with COVID-19 pneumonia: the CALL score. Clin Infect Dis.

[13] Knight S.R., Ho A., Pius R., Buchan I., Carson G., Drake T.M. (2020). ISARIC4C investigators. Risk stratification of patients admitted to hospital with covid-19 using the ISARIC WHO clinical characterisation protocol: development and validation of the 4C mortality score. BMJ.

[14] Liang W., Liang H., Ou L., Chen B., Chen A., Li C. (2020). Development and validation of a clinical risk score to predict the occurrence of critical illness in hospitalized patients with COVID-19. JAMA Intern Med.

[15] Mejia-Vilet J.M., Cordova-Sanchez B.M., Fernandez-Camargo D., Méndez-Pérez R.A., Morales-Buenrostro L.E., Hernández-Gilsoul T. (2020). A risk score to predict admission to intensive care unit in patients with COVID-19: the ABC-GOALS score. medRxiv.

[16] Weng Z., Chen Q., Li S., Li H., Zhang Q., Lu S. (2020). ANDC: an early warning score to predict mortality risk for patients with Coronavirus Disease 2019. J Transl Med.

[17] Jong VMT de, Rousset R.Z., Antonio-Villa N.E., Buenen A.G., Van Calster B., Bello-Chavolla O.Y. (2022). Clinical prediction models for mortality in patients with covid-19: external validation and individual participant data meta-analysis. BMJ.

[18] Marcolino M.S., Pires M.C., Ramos L.E.F., Silva R.T., Oliveira L.M., Carvalho R.L.R. (2021). ABC2-SPH risk score for in-hospital mortality in COVID-19 patients: development, external validation and comparison with other available scores. Int J Infect Dis.

[19] Lauring A.S., Tenforde M.W., Chappell J.D., Gaglani M., Ginde A.A., McNeal T. (2022). Influenza and Other Viruses in the Acutely Ill (IVY) Network. Clinical severity of, and effectiveness of mRNA vaccines against, COVID-19 from omicron, delta, and alpha SARS-CoV-2 variants in the United States: prospective observational study. BMJ.

[20] Brasil A. Nota técnica GVIMS/GGTES/ANVISA nº 07/2020 orientações para prevenção e vigilância epidemiológica das infecções por SARS-CoV-2 (COVID-19) dentro dos serviços de saúde. (complementar à nota técnica GVIMS/GGTES/ANVISA nº 04/2020). 2020.

[21] Vergouwe Y., Steyerberg E.W., Eijkemans M.J.C., Habbema J.D.F. (2005). Substantial effective sample sizes were required for external validation studies of predictive logistic regression models. J Clin Epidemiol.

[22] Nattino G., Finazzi S., Bertolini G. (2014). A new calibration test and a reappraisal of the calibration belt for the assessment of prediction models based on dichotomous outcomes. Stat Med.

[23] Landsheer J.A. (2016). Interval of uncertainty: an alternative approach for the determination of decision thresholds, with an illustrative application for the prediction of prostate cancer. PLoS One.

[24] Katikireddi S.V., Cerqueira-Silva T., Vasileiou E., Robertson C., Amele S., Pan J. (2022). Two-dose ChAdOx1 nCoV-19 vaccine protection against COVID-19 hospital admissions and deaths over time: a retrospective, population-based cohort study in Scotland and Brazil. Lancet.

[25] Thompson M.G., Natarajan K., Irving S.A., Rowley E.A., Griggs E.P., Gaglani M. (2022). Effectiveness of a third dose of mRNA vaccines against COVID-19-associated emergency department and urgent care encounters and hospitalizations among adults during periods of delta and omicron variant predominance - VISION network, 10 States, August 2021-January 2022. MMWR Morb Mortal Wkly Rep.

[26] Yek C., Warner S., Wiltz J.L., Sun J., Adjei S., Mancera A. (2022). Risk factors for severe COVID-19 outcomes among persons aged ≥18 years who completed a primary COVID-19 vaccination series ‒ 465 health care facilities, United States, December 2020-October 2021. MMWR Morb Mortal Wkly Rep.

[27] Liao D., Zhou F., Luo L., Xu M., Wang H., Xia J. (2020). Haematological characteristics and risk factors in the classification and prognosis evaluation of COVID-19: a retrospective cohort study. Lancet Haematol.

[28] Wang D., Hu B., Hu C., Zhu F., Liu X., Zhang J. (2020). Clinical characteristics of 138 hospitalized patients with 2019 novel coronavirus-infected pneumonia in Wuhan, China. JAMA.

[29] Petrilli C.M., Jones S.A., Yang J., Rajagopalan H., O'Donnell L., Chernyak Y. (2020). Factors associated with hospital admission and critical illness among 5279 people with coronavirus disease 2019 in New York City: prospective cohort study. BMJ.

[30] Bao C., Liu X., Zhang H., Li Y., Liu J. (2020). Coronavirus disease 2019 (COVID-19) CT findings: a systematic review and meta-analysis. J Am Coll Radiol JACR.

